# A Review of Self-Expanding Esophageal Stents for the Palliation Therapy of Inoperable Esophageal Malignancies

**DOI:** 10.1155/2019/9265017

**Published:** 2019-04-04

**Authors:** Yunqing Kang

**Affiliations:** ^1^Department of Ocean and Mechanical Engineering, Florida Atlantic University, Boca Raton, FL 33431, USA; ^2^Department of Biomedical Science, Florida Atlantic University, Boca Raton, FL 33431, USA; ^3^Integrative Biology PhD Program, Department of Biological Sciences, Florida Atlantic University, Boca Raton, FL 33431, USA

## Abstract

Esophageal cancer is a very deadly disease, killing more than 15,000 people in the United States annually. Almost 400,000 new cases happen in the worldwide every year. More than 50% esophageal cancer patients are diagnosed at an advanced stage when they need an esophageal stent to open the blocked esophagus for feeding and drinking. Esophageal stents have evolved in stages over the years. Current clinically used stents commonly include stainless steel or nitinol self-expandable metallic stent (SEMS) and self-expandable plastic stent (SEPS). There are many choices of different types of stents and sizes, with fierce competition among manufacturers. However, current stent technology, whether uncovered, partially covered, fully covered SEMS or SEPS, has their own advantages to solve the dysphagia, stricture, and fistula problems, but they also cause some clinical complications. The ideal stent remains elusive. New 3D printing technique may bring new promising potential to manufacturing personalized esophageal stents. Drug-eluting stents could be the new avenue to do more than just pry open a stricture or cover a defect in the esophageal lumen, a possibility of proving local anticancer therapy simultaneously. Additionally, the lack of esophageal cancer animal models also hinders the progress of stent development. This paper reviews these topics for a comprehensive understanding of this field. In a conclusion, the ultimate goal of the future esophageal stent would have multifunction to treat the underlying conditions and restore esophageal function to near normal.

## 1. Introduction

Esophageal cancer is a deadly disease, ranking sixth among all cancers in mortality [1]. About 17000 new esophageal cancer cases are diagnosed yearly and about 16000 deaths a year occur due to esophageal cancer in the United States (Cancer facts and figures. American Cancer Society). An estimated 456,000 new cases were diagnosed worldwide in 2012 [2], and there were over 500,000 new cases in 2018. More than 50% of esophageal cancer patients are diagnosed at an advanced stage when the esophagus has often been occluded by the tumor [3–5]. To open the occluded esophagus, a self-expanding esophageal stent is often used for drinking and feeding, which has become the primary palliative therapy of dysphagia [6]. Beyond just palliative therapy, researchers are exploring new therapeutic applications for an esophageal stent by wrapping a drug-eluting polymer film on the device [7, 8]. This wrapping polymer film provides the release of an anticancer drug to inhibit tumor growth. This kind of attempt in vascular stents has been proven to enhance treatments of blood vessel diseases [9, 10] and showed that it is feasible to treat both cancer-related stenosis as well as malignant gastrointestinal cancer [11]. This paper mainly focuses on reviewing the applications of self-expanding esophageal stents, biodegradable stent, and drug-eluting esophageal stents in malignant esophageal cancers, while the sizes, shapes, and manufactures of clinically used esophageal stents are not discussed as these aspects have been comprehensively reviewed in [6].

## 2. Self-Expandable Stents

### 2.1. Self-Expanding Metallic Stents (SEMS)

**Figure 1:**
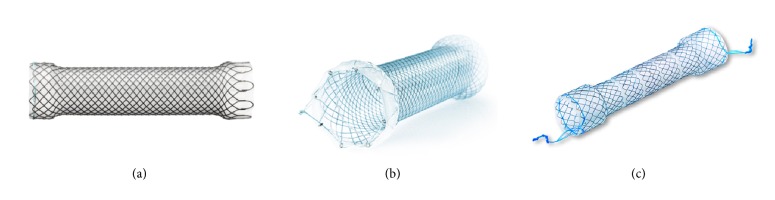
Photographs (from the company official websites) show Softcup esophagus SEMS stent (MICRO-TECH, Germany) (a), Permalume^TM^ Silicone coating SEMS (WallFlex^TM^ from Boston Scientifi, USA) (b), and a segmented SEMS (Choostent; M.I. Tech, Pyeongtaek, Korea) (c).

A variety of self-expanding esophagus stents have been widely used in the treatment of esophagus cancer [12, 13]. The esophagus stents include self-expanding plastic stents (SEPS) and self-expanding metal stents (SEMS). SEMS is the most widely used stents clinically for malignant esophageal cancer. Currently, SEMS are available in three main types: covered, partially covered, and uncovered (Figure 1). Although these types of SEMS have been widely used clinically, they cause complications differently. Multiple studies have reported that the conventional uncovered SEMS showed complications, such as bleeding, fistulae, recurrence of tissue growth, embedment, etc. [14]. The major complication is tissue regrowth to the mesh so that the stent is embedded in the tumor tissue, leading to new stricture or occlusion. Tumor ingrowth or overgrowth is one of the most delayed complications. In bare SEMS, about 17−36% showed tumor ingrowth [15]. Mayoral et al. analyzed the literature data of 81 patients receiving SEMS for esophageal cancer, and they found tumor overgrowth in 60% of the patients. Among these patients, 53% of the cases had a malignant growth while 47% had a nonmalignant growth over the stent [16]. To overcome the complications of tumor and granulation tissue ingrowth, a variety of covering polymer materials have been developed to fully cover the stent. However, after covering, new complications happened. The covered SEMS become prone to migration [17], leading secondary surgery to take out the stent from stomach. The most common complication of SEMS was migration (36.3%), followed by pain and obstruction [18]. To prevent the stent migration, the middle stem part of SEMS is covered but the proximal and distal ends of SEMS are exposed to allow embedding into the esophageal wall, thus helping to prevent the stent migration. An interesting method, through-the-scope (TTS) clips [19] or over-the-scope (OTS) [20], was developed to anchor the stent. Another interesting and promising method to anchor a stent to the esophageal wall is endoscopic suturing with an endoscopic suturing device [21]. To further review major advantages and drawbacks of SEMS, some representative SEMS of the most commonly used SEMS in the United States are listed in the table (Table 1). In general, these SEMS have common complications such as tumor tissue ingrowth and stent migration. The differences in migration rates among SEMS were not statistically significant [22].

### 2.2. Self-Expanding Plastic Stents (SEPS)

**Figure 2:**
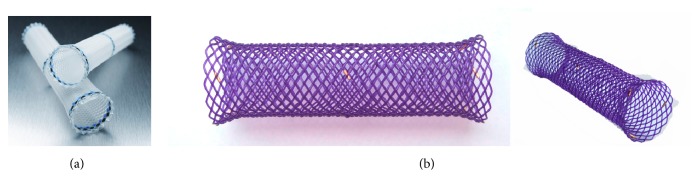
Photographs (from the company official websites) show the self-expanding plastic Polyflex stent (Boston Scientifi, USA) (a) and SX-ELLA degradable esophageal stent (ELLA-CS, s.r.o. Czech Republic) (b).

Although SEMS has been used clinically more than two decades, the rigid metal and large-sized ends occasionally cause patients' chest pain, bleeding, and fistulas or perforations [24, 25], even large tracheal-bronchial fistula [26]. Thus, the self-expanding plastic stents (SEPS) provides the advantages in mitigating these complications [27]. Further, SEPS can be easily removed and seems not to be inferior to metallic stent [28]. They also provide significant improvement of dysphagia and quality of life, and to decrease the number of dilatation sessions in patients with benign strictures [29]. The currently available SEPS is the Polyflex stent (Boston Scientific, Natick, MA, USA), which is composed of a plastic wire and has a fully covered design with silicone and a proximal flare (Figure 2(a)) This stent material has been suggested to reduce reactive tissue hyperplasia [30]. However, studies reported that SEPS have significantly higher rate of migration than SEMS. Holm and coworkers reported stent migration was more frequent in proximal (30/44 stents, 68.1%) and distal (19/27 stents, 70.4%) compared with mid esophageal (3/10 stents, 30%). Migration was more frequent in stents placed for benign strictures (18/22 stents, 81.8%), anastomotic strictures (18/24 stents, 75%), and fistulae/leak (13/22 stents, 59.1%) compared with radiation-induced strictures (4/14 stents, 28.6%) [31]. Although SEPS causes high rate of migration, it appears to be safer than metallic stent as the plastic material may not cause significant tissue trauma [32]. Therefore, these SEPS are often used in benign strictures, as well as for treating esophageal leaks, fistulae, and perforations. In a recent review paper, Gwang Ha Kim reviewed that SEPS were used in 130 patients with a benign esophageal stricture, and results showed that after a median follow-up period of 13 months, only 52% of the patients were dysphagia-free, the early migration rate was 24%, the endoscopic reintervention rate was 21%, and major clinical complications occurred in 9% of patients [30, 32].

In malignant management, SEPS became an alternative to SEMS since 2000s. Szegedi et al. compared the efficacy of SEPS and SEMS in palliation of advanced esophageal carcinoma [33]. They reported that the use of SEPS in palliation of esophageal cancer seems to be safer and more effective than SEMS in improving the quality of life of those patients. However, Conio and colleagues found that there was a significantly higher complication rate (hemorrhage, tumor overgrowth, and migration) with SEPS (Polyflex) compared to partially covered SEMS (Ultraflex) in 101 patients with unresectable carcinoma [34]. Twenty (44%) patients with a Polyflex SEPS stent and 18 (33%) with an Ultraflex SEMS stent had recurrent dysphagia because of tumor overgrowth, stent migration, hyperplastic granulomatous reaction, or food bolus impaction. Their results showed that more complications, especially late stent migration, were observed in the SEPS group.

### 2.3. Biodegradable Stents

To decrease the complications of tumor tissue or hyperplastic tissue growth and stent migration, and also to avoid the need for stent removal, new biodegradable stents (BD stent) have recently been developed. Two types of biodegradable polymer stents are available currently. One is the ELLA-BD stent (ELLA-CS, Hradec Kralove, Czech Republic), which is composed of polydioxanone, a surgical suture material (Figure 2(b)) [35], and the other one is the poly-L-lactic acid (PLLA)-BD stent (Marui Textile Machinery, Osaka, Japan), which consists of knitted PLLA monofilaments [23]. The advantage of biodegradable stent is that they do not have to be removed after they are implanted [36]. The gastric acid can hydrolysis the materials, which reduces the morbidity. Saito and colleagues developed Ultraflex-type stent by knitting poly-l-lactic acid (PLLA) monofilaments. They placed the biodegradable PLLA stent into 13 patients with different esophagus disorders including esophagus cancer. They found that no symptoms of restenosis were observed within the follow-up period of 7 months to 2 years. Further treatment with balloon dilatation or replacement of the biodegradable stent was not required [37]. However, spontaneous migration of the stents occurred between 10 to 21 d after placement in 10 of the 13 cases. The same research group continued to place the biodegradable PLLA stent into 2 cases with benign esophageal strictures. Their results showed that there were no symptoms of restenosis for 6 months. The PLLA esophageal stent provided a new possibility for the management of benign esophageal strictures after endoscopic submucosal dissection (ESD) [38]. van Boeckel PG compared the efficacy and safety of SEPS with the placement of biodegradable stents for the treatment of refractory benign esophageal strictures (RBES). Their results showed promising potential of the biodegradable stent for the treatment of RBES. They found that reinterventions were less using biodegradable stent than SEPS placement [39]. If using SEPS, twelve patients (67%) had recurrent dysphagia, but if using a biodegradable stent, only six patients (33%) have dysphagia. Obviously, biodegradable stents offered an advantage. However, biodegradable stents have also their drawbacks. Repici et al. conducted a prospective study in 21 patients using SX-Ella stents for RBES [40]. After 7 weeks, they reported that stent migration occurred in 9.5%. 45% of patients did not experienced dysphagia at the end of the follow-up period. However, 55% still suffered symptom recurrence of tissue ingrowth, and three patients experienced severe pain after placement. From these results, it can be seen that although biodegradable stents may provide a valuable alternative to SEPS and SEMS, and also may eliminate the need for repeat esophageal dilations, biodegradable stents still presented some complications of migration and tissue regrowth. Also, biodegradation may lead to the collapse of stents after placement due to the collapsed degradation of the stent, quickly losing the mechanical strength. For example, Nogales Rincon and colleagues placed a SX-ELLA esophageal degradable BD stent (ELLA-CS, Hradec Králové, Czech Republic) into a 74-year-old man with a previous history of surgery for pharyngolaryngeal neoplasia and reconstruction. They found that the stent degraded and collapsed after 9 weeks. The collapsed mesh did not allow the passage of a standard Pentax endoscope [41]. To overcome these issues of poor corrosion resistance and a quick loss of mechanical support of BD stents, Yuan et al. mixed biodegradable poly(*ε*-caprolactone) (PCL) and poly(trimethylene carbonate) (PTMC) as the coated membrane to coat magnesium alloy stents for a fully biodegradable esophageal stent. Their results showed that the new biodegradable stent had an ability to delay the degradation time and maintain mechanical performance in the long term [42, 43].

From these studies, it can be seen that the degradation properties of a BD stent determine its mechanical integrity. Studies showed that both ELLA-BD stent and PLLA-BD stents, the two currently available BD stents, can be degraded by hydrolysis, which is accelerated at low ambient pH. The stents began to degrade after 4 to 5 weeks and dissolved during a period of 2 to 3 months [23, 35]. The degradation rate of a BD stent is dependent not only on the properties of the polymer, but also the size and structure of the stent, and also influenced by surrounding environment, such as temperature, pH and type of body tissue/fluid. With time, these factors gradually affect the mechanical integrity of stent. Radial expansion force of stent would be also affected. The initial value of radial force of the current BD stent can be maintained in physiological saline solution (pH 7, 37°C) for 6-8 weeks. After seven weeks, the radial force was dramatically decreased and the stent was completely degraded after 2–4 months [44]. The polymer stent degraded faster with a lower pH. Therefore, in the future, when a new degradable stent will be designed, the polymer properties need to be optimized to achieve prolonged stent integrity. From these studies, due to the biodegradable features of the degradable stent, longer term studies are necessary to investigate the relationship between the expected disappearance of the stent and the patency of the management of esophagus disorders. The current evidence is insufficient to determine the relative efficacy or safety of esophageal biodegradable stents [45].

Thus, these esophageal stents had their own advantages and limitations. A fully covered SEMS decreases the recurrence of dysphagia associated with a bare SEMS, but it has a higher migration rate than a bare SEMS. Partially covered SEMS has a firm anchoring effect, preventing stent migration and the recurrence of dysphagia, but hyperplastic tissue reaction may easily happen. Likewise, SEPS reduces reactive hyperplasia, but the high rate of stent migration limited its application. A biodegradable stent brings an advantage of not requiring stent removal in comparison with SEMS and SEPS. However, it still has a somewhat high rate of hyperplastic reaction, and the risk of collapse, which does not satisfy expectations. Therefore, the question of which type of stent should be recommended for the effective treatment of complex and refractory benign strictures, also malignant tumor remains unclear [30].

### 2.4. 3D-Printed Stents

**Figure 3:**
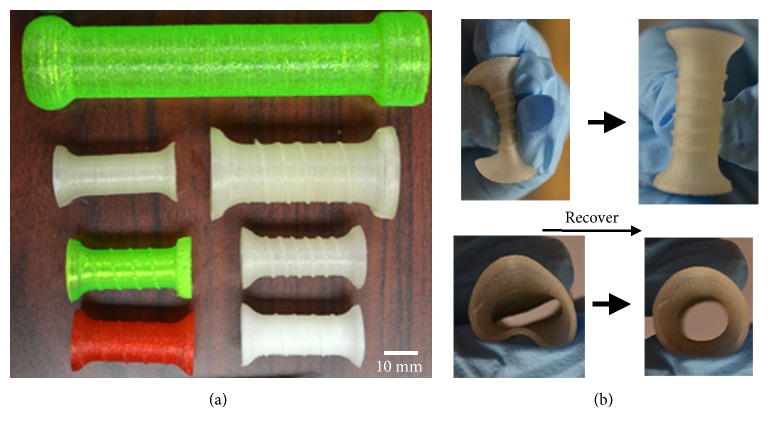
Photographs show the different types of 3D-printed stents with different structures and material ratios (a). The stent was compressed and then recovered to the original shape (b). Reedited and reprinted with the permission from [23].

More recently, additive manufacturing technologies have been used to fabricate stents, for example, airway stent [46]. Existing airway stents have many shortcomings including the development of obstructing granulation tissue in the weeks and months following placement, mucous build up within the stent, and cough. Furthermore, existing airway stents are expensive and, if improperly sized for a given airway, may be easily dislodged (stent migration). 3D-printing technology may offer the best chance of personalizing stents and addressing many of the current limitations in stent design. Similarly, 3D printing provides a new potential to produce esophageal stents. The 3D printing technology provides an advantage over the current traditional technologies for the preparation of polymer stents, such as braided, knitted, laser-cut [47], and segmented [48]. In our study, we used a 3D printing technology to produce a flexible polymer esophageal stent (Figure 3) [49]. We found that our 3D printing technique can print an esophageal stent with different size and shape. This is the first study using 3D printing technique to produce a polymer esophageal stent. Although the function of the 3D-printed flexible polymer stent has not been proved* in vivo*, the* in vitro* study showed that the 3D-printed esophageal stent has promising potential to treat malignant esophageal diseases. It can self-expand, and 3D printing technique can design and print different sizes and shapes of the stent easily. Further studies need to be done to further show the function of a 3D-printed esophageal stent.

### 2.5. Drug-Eluting Stents

For those patients with inoperable esophageal malignancies, using a SEMS to mechanically open the blocked esophagus for drinking and feeding has become the primary palliative therapy of dysphagia [6]. Recently, drug-eluting self-expanding metallic stents (DE-SEMS) have been attempted with some efficacy for the management of occlusion of the esophagus beyond just palliative procedure [7, 8]. DE-SEMS, a drug-loaded polymer membrane coated SEMS, provides both the mechanical support to expand the blocked esophagus, and the released anticancer drug to inhibit tumor growth, which has been proven to prolong patient life-span[11].

Cannular stents have been widely used in across a variety of vascular and nonvascular organs for unblocking the occlusion of body conduits. Drug-eluting stents (DESs) are popular for vascular applications, bile conduit [50], but the development of DES for nonvascular organ, for instance, esophagus, has been slow, and no drug-eluting stent is clinically available for treating esophageal cancer [51]. However, research on DESs for esophagus is following a similar track. Sanjay Garg et al. developed a bilayer polymer film loading an anticancer drug (docetaxel), which can be covered on a SEMS [52]. They used biocompatible, biostable polyurethane PurSil AL 20 (PUS) to prepare the docetaxel loaded films as a SEMS covering material for the localized delivery of DTX to the esophagus. They investigated the effect of thickness on release behavior and the permeation through esophageal tissues, thus establishing the critical factors responsible for controlling the delivery of DTX. They found that the bilayer films structure exhibited sustained release (>30 days) and minimal DTX permeation through esophageal tissues* in vitro*. They also found that the release rate lied with the esophageal tissues, suggesting that DTX delivery may be sustained for longer periods* in vivo* compared to the* in vitro*. This kind of localized sustained delivery system in combination with the stent appeared to be a promising strategy to treat malignant esophagus cancer. Fan et al. used rabbit esophageal cancer models to evaluate the efficiency and safety of paclitaxel-eluting SEMS [53]. Paclitaxel is currently being used to treat several types of cancer including esophageal carcinoma [54], through inhibiting tumor growth by binding to *β* -tubulin and stabilizing polymerized microtubules [50, 55]. Thus, paclitaxel-eluting SEMS (PEMS) may have antitumor effects against or prevent tumor overgrowth of malignant esophageal strictures through the local release to the esophagus tissue. They found that in the 22 rabbits, the average tumor volume was significantly decreased from 7.00±4.30 cm^3^ in the SEMS group to 0.94±1.51 cm^3^ in the drug-eluting stents group (p<0.05), and that the tumor area in the drug-eluting stent group was also smaller than that in the SEMS groups (p<0.05). This brought a promising clinical trial potential.

Besides loading the paclitaxel into a covering polymer film, other anticancer drugs, for instance, 5-fluorouracil were also loaded in the covering film of SEMS [56, 57]. The new development of this type of drug-eluting stent was a series of new films featuring multilayered structures for improved mechanical properties and unidirectional, controlled drug release [56, 58]. These studies aimed at solving the problem of drug leaking to the stomach through the cannula of stent and esophagus, because a drug-eluting film covering on a SEMS may lack the unidirectional drug release control to target the mucosa tissue of the esophagus [53]. Drugs from the thin film were released to the stomach through the mesh into the canal of the stent, which compromises the drug delivery efficacy of the drug-eluting SEMS and significantly increases side effects. To overcome this kind of complications, Tian et al. developed multilayered films based on a series of poly(caprolactone) (PCL) and PEG polymers, which contained antitumor 5-fluorouracil [58]. The covering film contained a backing layer, which blocks the release of anticancer drug to the stomach, and a surface multiple drug layers, which loaded different drug concentrations in different layers to realize unidirectional, controlled drug release. Their results showed that drug release was dependent on the drug loading and environmental pH, and that* ex vivo* permeation behaviors showed the drug released from the multilayered film in a unidirectional and controlled manner. This kind of the multilayered films provided an attractive mode to produce polymer covering stents for localized treatment of stenosis or occlusion of esophageal cancer. Similarly, Guo's research group recently developed a paclitaxel or 5-fluorouracil loaded bilayered polymer films covering on a nitinol SEMS to treat unresectable cancer in a porcine model [59, 60]. The bilayered polymer film is consisted of a layer of 50% PTX or 5-FU and a layer of drug-free backing. Their results showed that majority of the loaded drugs permeated into esophagus from the outside of the film, and the backing layer blocked the release of the drug to the cannula. The drug concentrations were highest in the esophagus compared with in the heart, liver, spleen, lung, kidney and blood (81500.0 ± 9475.2 ng/g versus 3.9 ± 0.3 ng/mL of PTX in the plasma at 13 days). This new stent provided a dual function as both a stent and a local drug delivery device for esophageal cancer.

A local, more targeted release of an anticancer drug from a bilayered or multilayered drug-releasing films demonstrated enhanced efficacy of anticancer drugs, prevented or reduced the side effects associated with systemic administration of an anticancer drug, such as paclitaxel, 5-fluorouracil, and gemcitabine [7, 11, 60–62]. Therefore, this type of drug-eluting stent has potential for improving inhibition of esophageal tumor growth.

## 3. Animal Models for Esophageal Stents

Currently, two-dimensional (2D) cell culture, 3D cell spheroids models, animal xenograft/orthotopic models are widely used in cancer studies to evaluate the efficacy and functionalities of a new drug delivery system or a new treatment [68–71]. Usually these models are feasible in interrogating the antitumor effect of drug delivery systems in many types of cancers [72, 73]. However, in esophageal cancers with strictures or benign esophageal diseases, these models encounter challenges for the studies of esophageal stents. This is because the esophageal stents commonly used in esophageal diseases include bared SEMS, SEPS, BD, covered stents, and drug-eluting stents. The length and diameter of most FDA-approved stents currently marketed in the United States are 8-10 cm and 16-20 mm [74], which is not applicable in a 2D cell culture, 3D cell spheroid model, or xenograft animal models due to the lack of sufficient dimensions and 3D esophageal biological structure [75–77]. For orthotopic esophagus models, stenting is still a challenge. For example, the esophagi of small animals, mice or rats, are too small to allow for stenting, compared to the deployment procedures designed for humans [78–80]. Rabbit models belong to moderate-to-large-sized animal models, but there are few reports of rabbit tumor models established for human-sized esophageal stents [81]. Large-sized animal models, such as the dog or pig, could simulate the human body environment for esophageal stent deployment, but most of studied dog or pig models used healthy esophagi to examine the safety of stenting and tissue responses. They were not established for esophageal cancer conditions [82, 83]. Therefore, there are very few appropriate animal esophageal cancer models available for the study of esophageal cancers and esophageal stents. There is an unmet need for the establishment of esophageal cancer animal models.

Although there are few orthotopic esophageal cancer models, in the past decades there are many reports about the use of animals for the test of esophageal stents* in vivo*. Shaikh et al. used a nude mice xenograft tumor model to test docetaxel (DTX)-loaded polyurethane formulations for stent application [84]. To test the esophageal tissue responses to nitinol stents loaded with 50% 5-FU or PTX, Guo et al. used twenty-three healthy Bama minipigs to test 4 groups for stent implantation: PTX stent, 5-FU stent, blank film–covered stent, and bare stent. They found that severe tissue responses including inflammation, ulceration, and granulation occurred at the bare ends of the stent not in the middle part of the stent. This animal model demonstrated that the drug concentrations in the esophagus that had contact with the 5-FU stent or PTX stent were very high, which did not cause obvious tissue damage [59, 60]. Chan Sup Shim et al. used the same model, minipig esophageal model, to test the clinical feasibility of a newly developed fully covered, self-expanding, through-the-scope (TTS) esophageal stent [85]. Besides these models, other animal models have been used to test drug-eluting stents, for instance, a dog esophagus stricture model was used to test the tissue response to a rapamycin-eluting nanofiber membrane-covered metal stent and a paclitaxel-eluting stent [86, 87], and a rabbit model was also used to test the efficacy of an IN-1233-eluting covered stent in preventing tissue hyperplasia [88]. A rabbit model was successfully used to test the efficiency of long-term local drug delivery of 5-fluorouracil-containing self-expandable nitinol stent [57].

These healthy animal models have been used to test the tissue safety of stents. However, a tumor model is required for evaluating the antitumor effect of self-expandable stent or drug-eluting stents that have been or are being developed. Very recently, Huang et al. successfully established a rabbit esophageal tumor model using endoscopic and surgical implantation of VX2 tumor fragments [81, 89]. Both the endoscopic and the surgical method had a relatively high success rate of tumor implantation [93.7% (30/32) versus 97.1% (33/34)] and tumor growth [86.7% (26/30) versus 81.8% (27/33)]. They continued to further evaluate the feasibility of the animal models for stenting [81]. The self-expandable metal stents were randomly deployed in rabbits with severe esophageal stricture to investigate the safety using the rabbit malignant model. The results indicated that the rabbits that received stent placement survived longer than those without stent implantation (the mean survival time: 53.9 days versus 40.3 days, p= 0.016).

To comprehensively review these reported models, a table is listed here to show the existing middle-to-large animal esophagus models for diverse purposes. All kinds of stents including SEMS, SEPS, BD, covered stent or new developed stents were listed in Table 2.

From Table 1 we can see that at present, only the rabbit malignant model is a middle animal esophagus tumor model for stent deployment, while most of other animal models used healthy esophagi or benign stricture esophagi. This is because it is challenging to establish a large animal esophageal cancer model. The challenge is not only because of the complex surgery on a large animal, but also the potential difficulty of inoculating tumor cells or tissue in the orthotopic esophagus for tumor formation. In the future, new animal models or alternative animal modelling technologies still need to be developed and established for esophageal cancer stenting.

## 4. Conclusion

Esophageal cancer remains a leading cause of cancer-related deaths worldwide. Palliation therapy for dysphagia using esophageal stents including SEMS, SEPS, and biodegradable stents is the current major treatment of choice for those patients with inoperable esophageal malignancies. Although these stents have been used in clinics for years, it is highly needed to develop novel stents that can overcome some complications associated with current stents. In addition to improving the functionality of the drug-loaded stent with markedly reduced adverse effects, new ideal stents will allow to be tailored to individual needs at much lower cost. Additionally, there is an unmet need to develop a large animal esophageal cancer model* in vivo* and establish a functional esophageal cancer model* in vitro* to test stents and study esophageal cancers.

## Figures and Tables

**Table 1:** Overview of advantages and drawbacks of some SEMS commonly used in clinics.

Product	Manufacturer	Advantages	Drawbacks	Techniques to prevent migration

Alimaxx-ES EndoMAXX	Merit Medical	(i) Laser cut designed to meet specific anatomical requirements. (ii) Polyurethane cover helps decrease tissue ingrowth. (iii) Silicone lining provides a smooth inner lumen. (iv) Purse-string design of the proximal suture knot allows repositioning and removal immediately post-placement.	(i) One case report that the patient presented with vomiting and dysphagia to solids [63].	(i) Anti-migration struts design reduces stent migration.
Choo stent	M.I. Tech	(i) Fully covered with polyurethane to prevent tumor ingrowth. (ii) Cylindrical zigzag fashion of 12 or 15 bends to maximize its longitudinal flexibility. (iii) Easily removed under mild sedation.	(i) The distal release mechanism may cause inconvenience in placement [64].	(i) Distal flared extremity.
Niti-S Double-layered stent	Taewoong Medical	(i) Prevent tumor ingrowth and migration.	(i) May cause overgrowth at the proximal end of the stent [65].	(i) Stent-inside-a-stent.
SX-ELLA-HV	Ella-CS	(i) The ends are non-traumatic. (ii) Covered film prevents tumor in-growth and occlusion. (iii) Unique delivery system.	(i) The frequency of hemorrhage and fistula formation was considerable. (ii) It has similar migration rate to other fully covered stents [66].	(i) Collar anti-migration system.
Evolution	Cook Medical	(i) Silicone encases the exterior and interior surfaces of the stent to prevent tumor ingrowth. (ii) A dual purse string “lasso loop” on the proximal and distal ends of the stent facilitates repositioning. (iii) Unique delivery system.	(i) Potential risk of aspiration [67].	(i) Proximal and distal uncovered flares.
Ultraflex/Wallflex	Boston Scientific	(i) A purse string facilitates stent repositioning or removal.	(i) Potential risk of bleeding [67].	(i) Progressive step flared ends reduces migration.

**Table 2:** Current animal esophagus models for stenting.

Animal model	Inserted stent	Purposes	Results	Refs

Healthy rabbit	SEMS with ^125^I loaded	To evaluate radiotolerance	Caused epithelial hyperplasia and stricture	[90]
Canine stricture	New covered SEMS	To test the antimigration	Half of stents migrated	[91]
Mongrel dogs	New nitinol stent	Anti-postcaustic stricture	Better than unstented group	[92]
Bama mini-pig	Nitinol stents loaded 5-FU or Paclitaxel (PTX)	To investigate tissue response; Drug release	Severe tissue response at the ends; highest drug concentrations in esophagus	[59, 60]
New Zealand rabbits	magnetocaloric nitinol stent with PTX	Drug eluting Release	biocompatible and safe	[93]
Healthy beagle dogs	Covered SEMS	Evaluate safety	No significant radiation toxicity	[94]
Benign dog cardia stricture	paclitaxel or rapamycin-eluting stent	Observe inflammatory reaction	Drug-eluting stent had better outcomes	[95]
A stricture model of rabbit	Three “piece” of SEMS with PLGA treads	Safety of the stent	The degradable part of the stent degraded; stent migrated	[96, 97]
Mini pig	Full covered SEMS	to evaluate the clinical feasibility	Easy deployment;	[82, 98]
Refractory benign strictures in dogs	SEMS, SEPS, BD	To evaluate the complications	50% dogs had complications	[99]
Pig stricture model	ELLA-CS); PLA/PCL BD stent	To treat stricture	Did not prevent high-grade stricture formation.	[100, 101]
Rabbit model.	IN-1233–eluting covered stents	To investigate the efficacy	decreased tissue hyperplasia	[88]
Dog model	PCDL BD stent	To treat stenosis	The stent recovered its initial shape in vivo	[102]
Malignant rabbit models	SEMS, drug-eluting stent	To image cancer tissue, and treat	Successful in establishing a malignant esophagostenosis model in rabbits	[7, 81, 103]
